# The effect of dentin surface pretreatment using dimethyl sulfoxide on the bond strength of a universal bonding agent to dentin

**DOI:** 10.1186/s12903-023-02913-3

**Published:** 2023-04-29

**Authors:** Kianoosh Mirzaei, Elham Ahmadi, Niyousha Rafeie, Mahdi Abbasi

**Affiliations:** 1 Department of Operative Dentistry, School of Dentistry, Semnan University of Medical Sciences, Semnan, Mirzaei Iran; 2 Dental Research Center, Dentistry Research Institute, Department of Restorative Dentistry, School of Dentistry, Tehran University of Medical Sciences, North Kargar, Tehran, 14174 Iran; 3 Dental Research Center, Dentistry Research Institute, School of Dentistry, Tehran University of Medical Sciences, Tehran, Iran

**Keywords:** Dimethyl sulfoxide, Universal adhesive, Bond strength, DMSO, Microleakage, Self-etch, Total-etch

## Abstract

**Background:**

This study aimed to evaluate the effect of dentin pretreatment by Dimethyl Sulfoxide (DMSO) on the bond strength and microleakage of a universal bonding agent to dentin.

**Methods:**

Fifty-six dentinal discs (thickness = 2 mm) were obtained from the crowns of the human third molars. The disks were assigned into 4 groups and treated as follows; self-etch-control group: G-Premio universal adhesive was used in self-etch mode, total-etch-control: G-Premio universal adhesive was used in total-etch mode, self-etch-DMSO: Water-based DMSO (50% volume) was applied on the samples for 60 s followed by application of G-Premio universal adhesive in self-etch mode, and Total-etch-DMSO: The samples were etched, and then, water-based DMSO was applied on them for 60 s followed by the application of G-Premio universal adhesive in total-etch mode. Afterward, resin composite was placed on all samples and light-cured. The samples were kept in distilled water and subjected to 5000 thermal cycles. Microshear bond strength was measured using the universal testing machine and failure modes were analyzed using a stereomicroscope. Forty-eight human third molars were used for microleakage evaluation and a standardized class five cavity was prepared on the buccal surface of each tooth. The teeth were assigned into 4 groups and received aforementioned surface treatment and the cavities were filled with resin composite. After storing in water for 24 h, the samples were subjected to 5000 cycles of thermocycling and the microleakage level of the samples was evaluated using silver nitrate uptake at the bonded interface. Two-way ANOVA test was used to analyze the effect of bonding technique (self-etch/ total-etch) and DMSO pretreatment on the microshear bond strength and microleakage of G-Premio adhesive to dentin.

**Results:**

Bonding technique had no effect on the bond strength values (p = 0.17) while DMSO pretreatment significantly decreased the microshear bond strength of the samples (p = 0.001). DMSO application increased microleakage significantly in total-etch (P-value = 0.02) while it had no effect in self-etch mode (P-value = 0.44).

**Conclusions:**

Pretreatment of dentin using 50% DMSO significantly reduced the bond strength of G-Premio Bond in both self-etch and total-etch modes. DMSO effect on microleakage depended on the etching technique; DMSO increased the microleakage level when the adhesive was used in total-etch mode while did not affect the microleakage in self-etch mode.

**Supplementary Information:**

The online version contains supplementary material available at 10.1186/s12903-023-02913-3.

## Background

In recent decades, resin composite restorative materials have been increasingly used in dental practice. These materials have become popular among dentists as well as patients due to their esthetic properties fulfilling patients’ demands. Moreover, these restorative materials allow practitioners to preserve more tooth structure during cavity preparation. This conservative cavity design relies significantly on the adequate bond between dentin and the bonding agent [1]; however, durability of this bond has been a challenging topic in adhesive dentistry. It is believed that excessive residual water on the dentin surface contributes significantly to the limited durability of the resin-dentin bond by three possible mechanisms: First, water allows endogenous matrix metalloproteinases (MMPs) and cysteine cathepsins to degrade unprotected dentin collagen fibrils which in turn, inhibits sufficient resin bonding impregnation into dentin [2] and subsequently decreases the bond strength of resin composite to dentin. Decreased bond strength is responsible for possible future problems such as leakage, recurrent caries, and loss of the restoration and tooth structure over time [3]. Second, water causes phase separation in adhesive components which in turn, contributes to the hydrolytic degradation of the adhesive resin. Third, water accelerates the hydrolysis of the polymers containing ester linkages [4]. As a result, various approaches have been proposed to overcome these challenges and improve the limited durability of the resin-dentin bond. Ethanol wet bonding is one of these approaches which aims to remove water from the exposed dentinal collagen and replace it with more hydrophobic components of the resin. Excluding water using high ethanol concentrations might decrease the hydrolytic degradation of dentinal collagen fibers as well as resin components presented in the resin-dentin hybrid layer. According to the previous studies [5–7], the ethanol-wet bonding technique showed satisfactory results when used in conjunction with hydrophobic adhesives. However, despite its advantages, this method is not clinically feasible because of technique sensitivity and being time-consuming. Other proposed techniques do not sufficiently address the problem of hydrolytic degradation of the adhesive resin and collagen fibers concurrently [2].

Recently, the application of dimethyl sulfoxide (DMSO) to improve dentin-resin bond has gained researchers’ attention; this compound is a polar aprotic solvent with a high ability to penetrate biological surfaces due to its small size, dipolar aprotic nature [8], and capability of dissolving both polar and non-polar compounds [3]. Previous studies have reported that DMSO application can improve the immediate and long-term bond strength of resin to dentin since DMSO can dissociate the highly cross-linked collagen fibers into a sparser network, improving resin diffusion into the collagen matrix [8, 9]. Moreover, DMSO can dissociate residual water on the dentin surface and thus, improve the wettability of demineralized dentin [4]. It also hinders collagen degradation by inhibiting MMP 9 and MMP 2 enzymes [10].

Previous studies have mostly evaluated the effect of DMSO pretreatment on the bond strength of 2 or 3-step adhesive systems and there is a controversy in the results; Stape et al. [2] evaluated the effect of DMSO dentin pretreatment on the bond strength of self-etch and total-etch adhesives to dentin and reported that DMSO pretreatment significantly improved the bond strength. On the other hand, according to Tjäderhane et al. [8], dentin pretreatment using DMSO could not significantly change the bond strength of an etch-and-rinse adhesive to dentin after 24 h. Mello et al. investigated the effect of DMSO application on the microtensile bond strength of a universal adhesive used in self-etch and total-etch mode and reported DMSO application did not affect bond strength to dentin even after 6 months [11].

In addition to bond strength, microleakage is an important factor for evaluating the sealing ability of the material and the quality of the hybrid layer formed between dentin and adhesive that affects the longevity of adhesive-based restorations [4].

Due to the inconsistent results of the previous studies and the fast, easy, and less technique-sensitive application of universal adhesives, the present study aimed to investigate the effect of dentin pretreatment with DMSO on the microleakage and bond strength of a universal adhesive to dentin. The null hypothesis was that pretreatment of dentin with DMSO would have no effect on the microshear bond strength and microleakage of a universal bonding adhesive agent to dentin.

## Methods

### Sample size

In this *in-vitro* study (ethics code: IR.TUMS.DENTISTRY.REC.1399.171), the minimum sample size for bond strength evaluation was calculated to be 14 samples in each experimental group according to a similar study [12], using One-way ANOVA feature of PASS 11 software (NCSS, LLC., Kaysville, Utah, USA), considering alpha = 0.05, beta = 0.2, standard deviation equal to 4 MPa, and the effect size of 0.46.

For microleakage evaluation, the minimum sample size was calculated to be 12 specimens in each experimental group according to a similar study conducted by Abaza et al. [13], using One-way ANOVA feature of PASS 11 software (NCSS, LLC., Kaysville, Utah, USA), considering alpha = 0.05, beta = 0.2, and standard deviation of 0.63 and effect size of 0.50.

### Sample preparation

One-hundred and four intact human third molar teeth without cracks and caries extracted for surgical reasons were used in this study. The teeth were cleaned using ultrasonic cleaner and disinfected in 1% chloramine-T solution at 4 °C for one week [14] and then, were stored in distilled water at 4 °C until use. Next, fifty-six teeth were randomly selected to be used for microshear bond strength evaluation. The teeth were mounted in acrylic cylindrical molds and the roots were cut 1 mm below the CEJ using diamond disks. The occlusal enamel was also grounded to expose dentin. Then, the dentinal disks with 2 mm thickness were obtained from the middle third of the teeth. To standardize the surface roughness, the dentinal disks were polished using 600 grit silicon carbide sandpapers (Diamond Pro, FGM, Brazil) under water irrigation [15]. The samples, then, were randomly divided into 4 groups and received treatment as follows:

- Self-etch-control (SE-C): The samples were dried and the bonding agent (G-Premio Bond universal adhesive, GC Corp., Tokyo, Japan) was placed on the samples. After 10 s, the bonding agent was dried with dry air for 5 s followed by 20 s of light-curing with a light-cure device (Woodpecker LED Curing, Guilin Woodpecker Medical Instrument Co., Guilin, China) with 1000 mW/cm^2^ of power.
- Total-etch-control (TE-C): The samples were dried and etched with 37% phosphoric acid (Condac 37% phosphoric acid etching gel, FGM Dental Group, Santa Catarina, Brazil) for 15 s, and then, were rinsed for 15 s and dried gently. The bonding agent was placed on the samples, and after 10 s, it was dried with dry air for 5 s followed by light-curing for 20 s with the light-cure device.
- Self-etch-DMSO (SE-DMSO): Water-based DMSO (50% volume) (Merck KGaA, Darmstadt, Germany) was applied on the samples with light-pressure circular scrubbing movements for 60 s, using a disposable microbrush (FGM Dental Group, Santa Catarina, Brazil). Next, the bonding agent was placed on the samples, and after 10 s, the bonding agent was dried with dry air for 5 s followed by 20 s of light-curing with the light-cure device.
- Total-etch-DMSO (TE-DMSO): The samples were dried and etched with 37% phosphoric acid for 15 s, and then, were rinsed for 15 s and dried gently. Next, water-based DMSO (50% volume) was applied on the samples with light-pressure circular scrubbing movements for 60 s. Afterward, the bonding agent was placed on the samples, and after 10 s, the bonding agent was dried with dry air for 5 s followed by 20 s of light-curing with the light-cure device.

In the end, a tygon tube (diameter of 1.2 mm and height of 1 mm) was placed on each sample and filled with Filtek Z250 resin composite (3 M ESPS, St Paul, Minnesota, USA) by the incremental technique, followed by 20 s of light-curing.

The trade name, manufacturer, and composition of the used materials are summarized in Table 1.

**Table 1:** Trade name, manufacturer, and composition of the used materials in the present study

Trade name (Manufacturer)	Composition
Filtek Z250 resin composite (3 M ESPS, St Paul, Minnesota, USA)	Silane Treated Ceramic, Bisphenol A Polyethylene Glycol Diether Dimethacrylate, Aluminum Oxide, Diurethane Dimethacrylate (UDMA), Bisphenol A Diglycidyl Ether Dimethacrylate (Bis-GMA), Triethylene Glycol Dimethacrylate (TEGDMA)
G-Premio Bond universal adhesive (GC Corporation, Tokyo, Japan)	10-MDP, 4-META, 10-Methacryoyloxydecyldihydrogen Thiophosphate, Methacrylate Acid Ester, Distilled Water, Acetone, Photo-initiators, Silica Fine Powder
Dimethyl Sulfoxide (Sigma–Aldrich Co., St. Louis, MO, USA)	C_2_H_2_OS

### Aging procedure

The samples were stored in distilled water for 24 h at 37 °C to ensure complete polymerization of resin composites. Afterward, the samples were subjected to 5000 cycles of thermocycling (TC3000, Vafai Industrial Co., Tehran, Iran) in water baths between 5 and 55 °C with a dwell time of 20 s which corresponds to 6 months of function in the oral cavity [16].

### Microshear bond strength evaluation

The samples were de-bonded using a universal testing machine (Z050 model, Zwick GmbH, Ulm, Germany) at a cross-head speed of 1 mm/minute and the microshear bond strength values were calculated according to the following formula:

1 $$\displaylines{Microshear{\text{ }}bond{\text{ }}strength{\text{ }}(MPa) = \cr \frac{{Force{\text{ }}(N)}}{{Surface{\text{ }}area{\text{ }}(m{m^2})}} \cr}$$

Bond failure sites were examined using a stereomicroscope (SMZ 800 model, Nikon Co., Tokyo, Japan) under × 40 magnification in order to determine the modes of failure. Failure modes were classified as follows:

Adhesive mode: Failure in the dentin-resin composite interface.

Cohesive mode: Failure within the dentin or resin composite bulk.

Mixed mode: Combination of both adhesive and cohesive failures.

### Microleakage evaluation

Forty-eight remaining teeth were used for microleakage evaluation. A standardized class 5 cavity (3×3×2 mm) was prepared on the buccal surface of each tooth 1 mm above the CEJ. A pear-shaped diamond bur (822, Komet Co., South Carolina, USA) was used in a high-speed handpiece to prepare the cavities. To standardize the cavity’s dimensions, a rectangle was cut on a matrix band and was used during cavity preparation. The depth of the cavities was measured using a periodontal probe.

The teeth were randomly assigned into 4 groups and received surface treatments as previously mentioned.

After surface treatment, resin composite (3 M ESPS, St Paul, Minnesota, USA) was placed in the cavities with incremental technique and light-cured for 20 s. The restored cavities were polished with Diamond Pro polishing disks (FGM Dental Group, Santa Catarina, Brazil). The samples were stored in distilled water at 37° C for 24 h to ensure complete polymerization. Afterward, the samples were subjected to 5000 cycles of thermocycling (TC3000, Vafai Industrial Co., Tehran, Iran) in water baths between 5 and 55 °C with a dwell time of 20 s which corresponds to 6 months of function in the oral cavity [16].

**Fig. 1:**
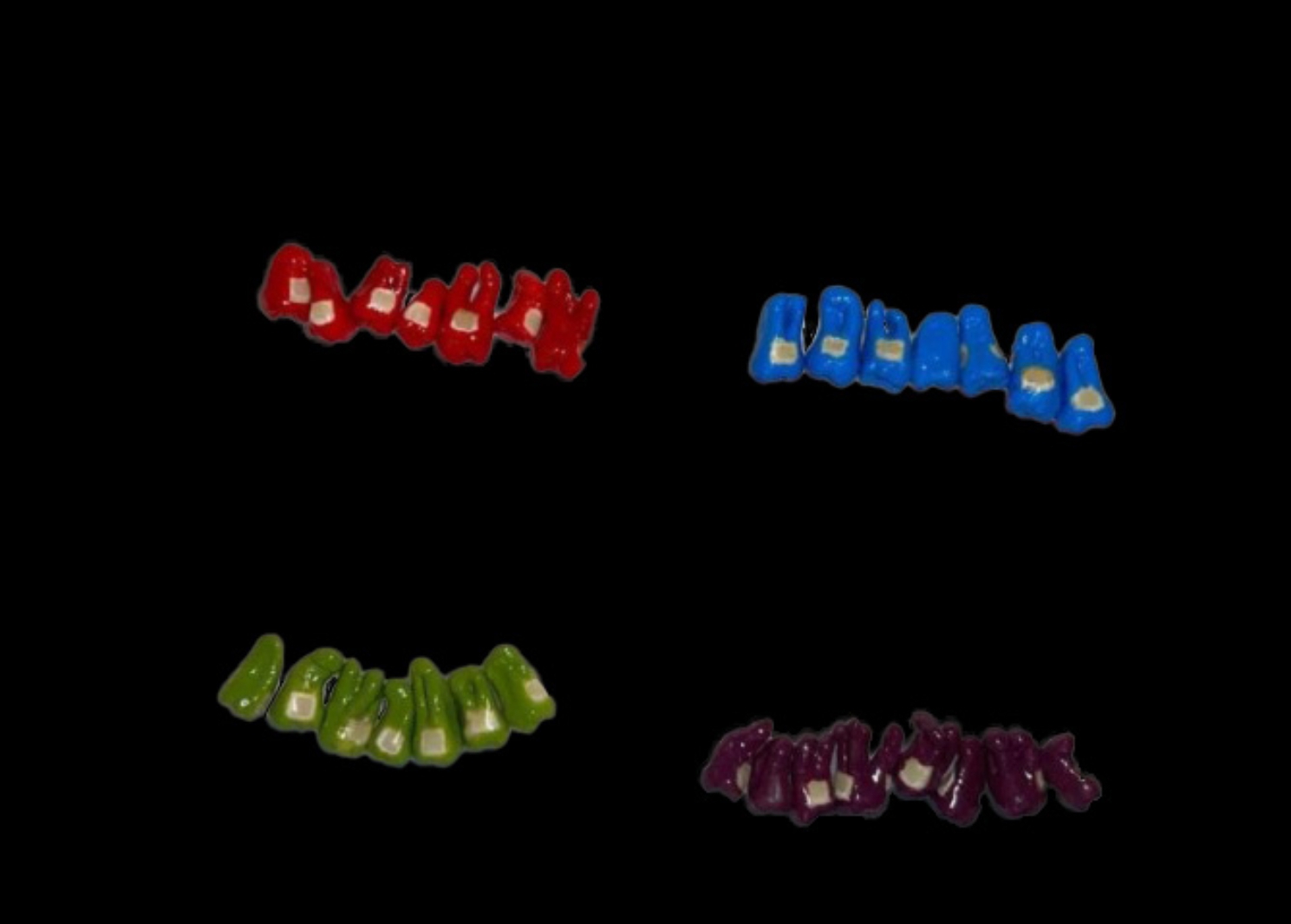
Samples were coated with two layers of nail varnish applied up to 1 mm of the bonded interfaces. Different colors were used to mark different experimental groups

The microleakage of the samples was evaluated using silver nitrate uptake at the bonded interface. For this purpose, the samples were coated with two layers of nail varnish applied up to 1 mm of the bonded interfaces (Fig. 1). Then, the samples were stored in 1 M silver nitrate solution (Sigma–Aldrich Co., St. Louis, USA). After 6 h, the samples were thoroughly rinsed with distilled water, and were immersed in a photo-developing solution for 8 h under fluorescent light to limit the penetration of silver ions into metallic silver grains along the bonded interface [2]. The samples, then, were cut buccolingually with a low-speed diamond saw in a cutting machine with water coolant. The microleakage was evaluated with a stereomicroscope. The Photoshop software was used to measure the length of the occlusal, axial and gingival walls of the preparation. The corresponding lengths of microleakage occurring along each wall were also measured. The microleakage level for each sample was defined as the sum of the lengths of penetrated silver nitrate along the walls divided by the sum of the lengths of the occlusal, axial, and gingival walls [17, 18].

### Statistical analysis

Data analysis was performed using Two-way ANOVA in SPSS software (SPSS Inc., Chicago, IL, USA). The level of significant difference was considered less than 0.05 (P-value < 0.05).

## Results

### Microshear bond strength

The mean and standard deviation of microshear bond strength values are summarized in Table 2. The highest microshear bond strength was observed in the self-etch-control group (SE-C) followed by total-etch-control (TE-C), self-etch-DMSO (SE-DMSO), and total-etch-DMSO (TE-DMSO) respectively.

**Table 2:** Descriptive statistics of microshear bond strength values (MPa), frequency, and percentage (%) of failure modes in different experimental groups

Pretreatment with DMSO	Mean ± SD	Minimum	Maximum	Frequency of failure mode		
				Mixed	Adhesive	Cohesive
SE-C	33.04 ± 8.17	19.14	44.61	8 (57.1%)	6 (42.9%)	0
SE-DMSO	25.03 ± 8.98	13.84	35.93	6 (42.9%)	8 (57.1%)	0
TE-C	29.09 ± 8.22	18.44	46.06	5 (35.7%)	9 (63.3%)	0
TE-DMSO	23.55 ± 5.27	16.07	33.07	6 (42.9%)	8 (57.1%)	0

**Fig. 2:**
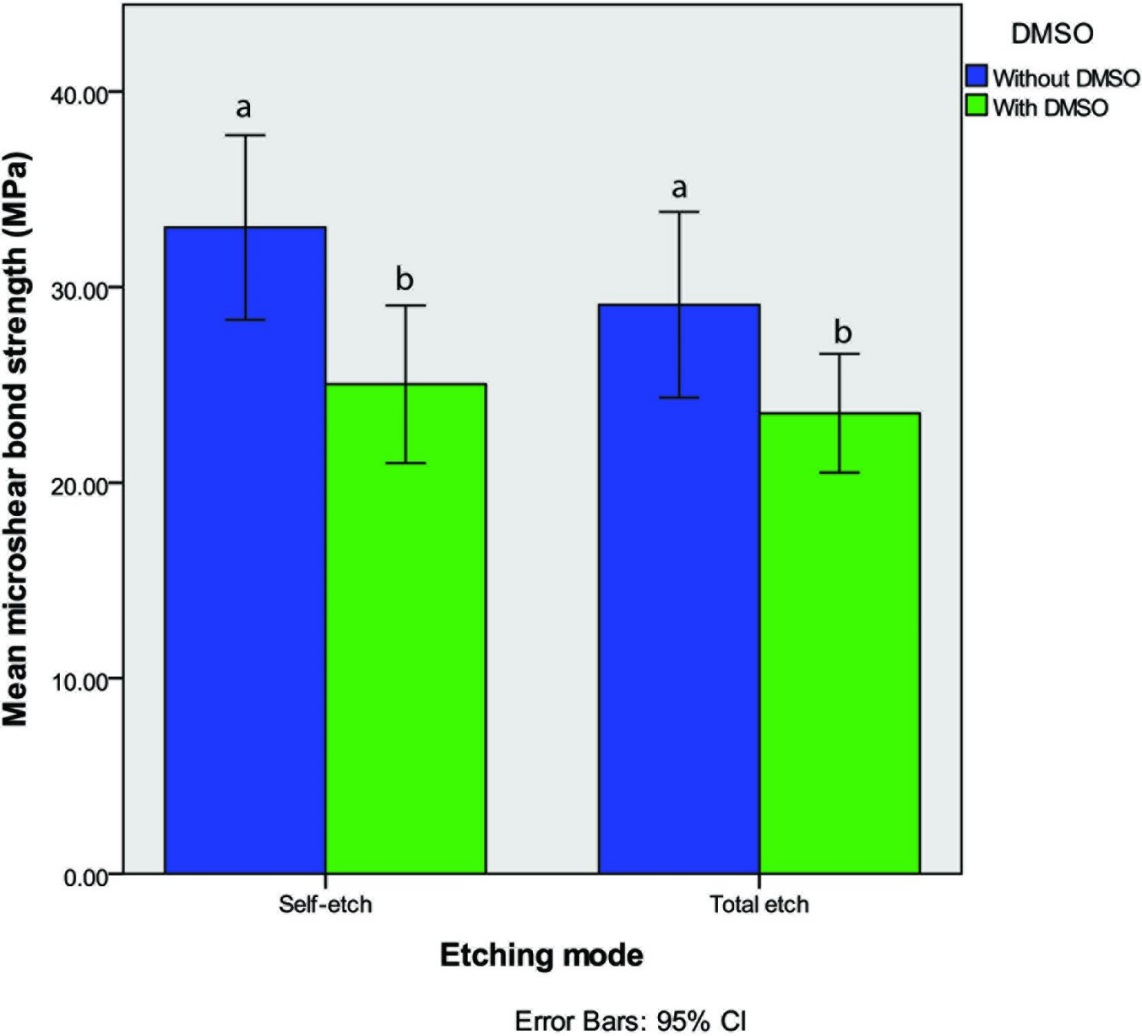
Mean and 95% confidence interval (CI) of the microshear bond strength values in self-each and total-etch groups with and without DMSO pretreatment. Different lowercase letters show significant difference between the groups

Two-way ANOVA analysis revealed that microshear bond strength of samples in self-etch groups did not significantly differ from those of total-etch groups (P-value = 0.167). In fact, the bonding technique (self-etch/ total-etch) did not affect the microshear bond strength values significantly. However, dentin pre-treatment with DMSO decreased the bond strength in both the self-etch and total-etch groups (0.001) (Fig. 2). No interaction was observed between the bonding technique and DMSO pretreatment (P-value = 0.52).

**Fig. 3:**
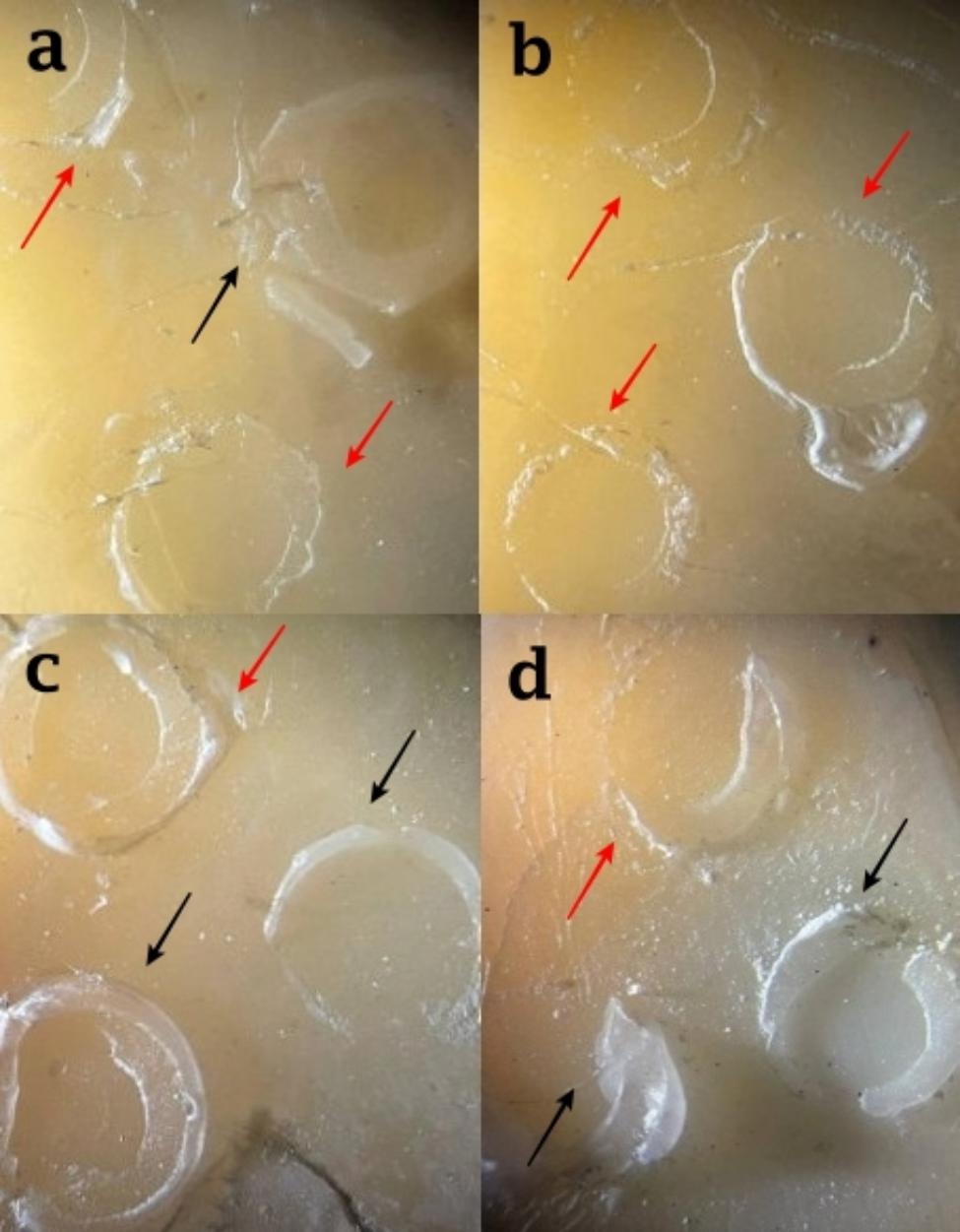
Failure modes in (a) total-etch group with DMSO pretreatment, (b) total-etch group without DMSO pretreatment, (c) self-etch group with DMSO pretreatment, and (d) self-etch group without DMSO pretreatment. Red and black arrows show adhesive and mixed failure patterns respectively

In SE-C group, the number of mixed failures was higher than adhesive failures while in SE-DMSO, and both total-etch groups, the adhesive failure was more frequent than the mixed failure. The cohesive mode was observed in none of the groups (Table 2). Figure 3 illustrates frequent modes of failure observed in different experimental groups.

### Microleakage

The highest and lowest silver uptake was observed in TE-DMSO and TE-C groups respectively (Table 3). According to the Two-way ANOVA results, the bonding technique (P-value = 0.54) and DMSO application (P-value = 0.15) did not have a significant effect on the microleakage values while their interaction had a significant effect on the microleakage expression (P-value = 0.01).

**Table 3:** Descriptive statistics of microleakage levels in experimental groups

Group	Mean ± SD	Minimum	Maximum
SE-C	33.33 ± 9.91	20.32	51.16
SE-DMSO	29.76 ± 12.41	15.12	47.03
TE-C	22.63 ± 7.98	15.28	43.25
TE-DMSO	36.27 ± 16.02	17.90	64.31

Regarding the bonding technique, in the groups in which DMSO was not used, the microleakage level was significantly higher in self-etch compared to total-etch mode (P-value = 0.008). However, when DMOS was applied, no significant difference was observed between self-etch and total-etch groups (P-value = 0.28).

**Fig. 4:**
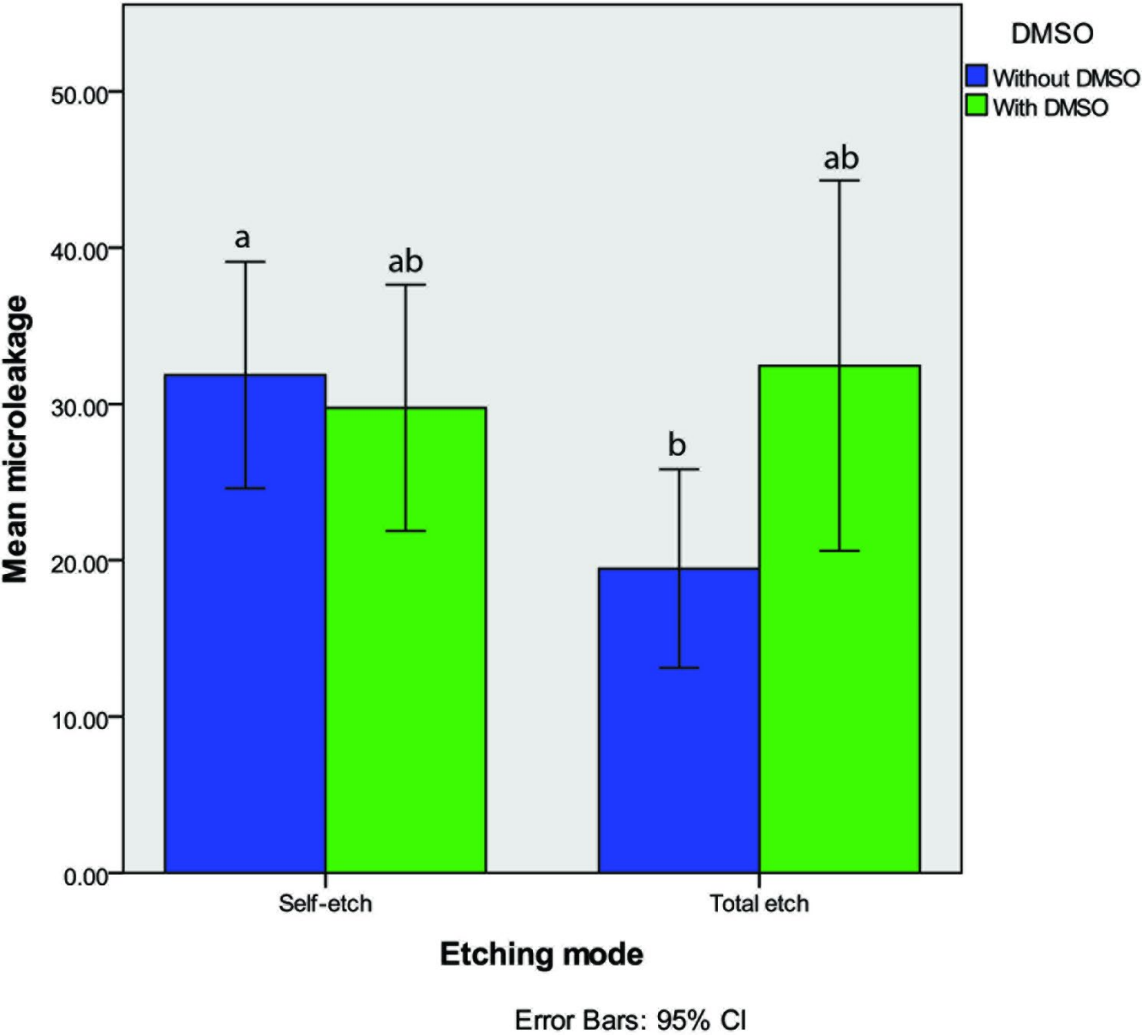
Mean and 95% confidence interval (CI) of the microleakage values in self-each and total-etch groups with and without DMSO pretreatment. Different lowercase letters show significant difference between the groups

**Fig. 5:**
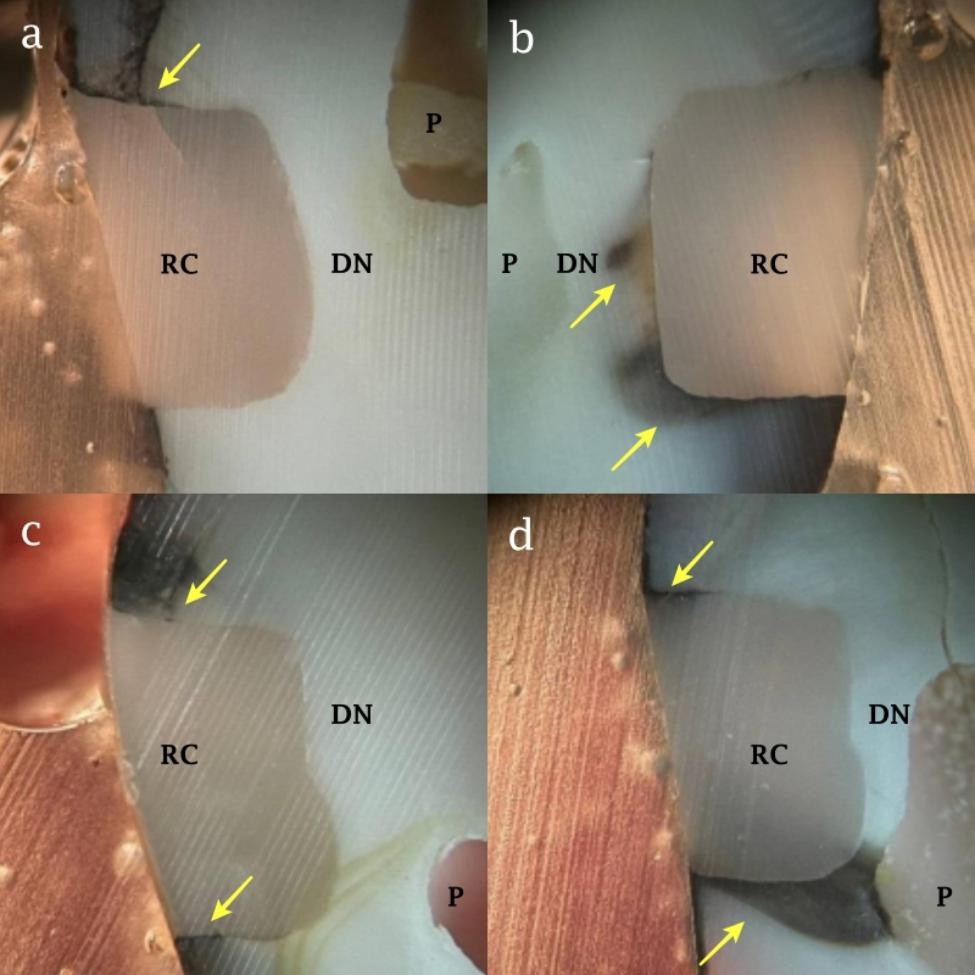
Microleakage in total-etch groups, (a) microleakage in DMSO treated sample. Microleakage is seen in occlusal wall of the cavity (yellow arrow), (b): microleakage in another DMSO treated sample. Microleakage is seen in gingival and axial walls of the cavity (yellow arrow), (c) and (d): microleakage in the sample without DMSO treatment. Microleakage is seen in both occlusal and gingival walls of the cavity (yellow arrow). RC: resin composite, DN: dentin, and P: pulp chamber

**Fig. 6:**
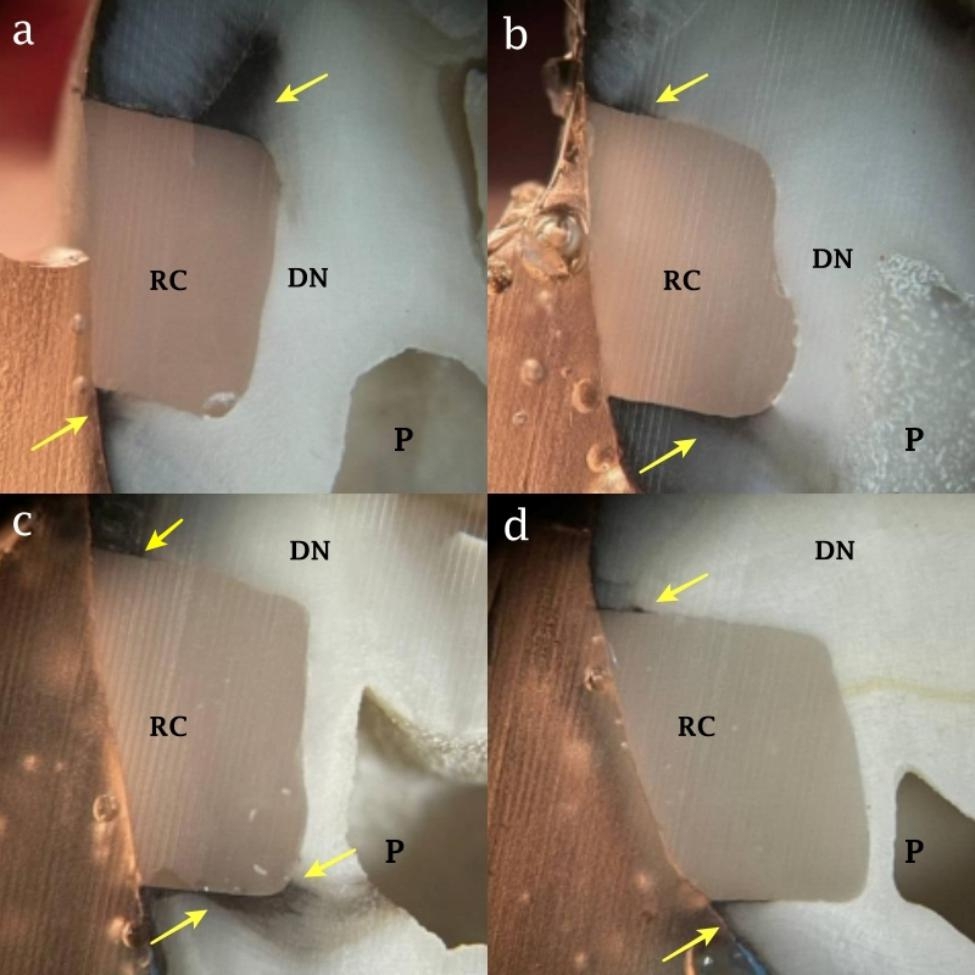
Microleakage in self-etch groups, (a) and (b) microleakage in DMSO treated samples. Microleakage is seen in both occlusal and gingival walls of the cavity (yellow arrow), (c) and (d) microleakage in the samples without DMSO treatment. Microleakage is seen in both occlusal and gingival walls of the cavity (yellow arrow). RC: resin composite, DN: dentin, and P: pulp chamber

Regarding the DMSO application, DMSO increased the microleakage level in total-etch group significantly (P-value = 0.02) while no significant difference was observed in the microleakage levels of self-etch group with this regard (P-value = 0.44). Figure 4 shows the mean and 95% confidence interval of the microleakage values in different experimental groups. The stereomicroscope images of microleakage in total-etch and self-etch groups with and without DMSO pretreatment are presented in Figs. 5 and 6.

## Discussion

Bonding of resin adhesive to dentin has remained challenging despite considerable advances in dental adhesion science in recent years [19, 20]. Dentinal collagen fibrils act as a scaffold for resin infiltration to form a hybrid layer. However, this hybrid layer is referred to as the weakest part in the resin-dentin interface [21]. Several approaches have been proposed to improve the quality of this hybrid layer, among which dentin pretreatment with DMSO has shown promising results. DMSO might improve resin-dentin bonding by the following mechanisms; (1) Bonding to dentinal proteins, (2) increasing interspacing of collagen fibrils, (3) facilitating monomer penetration into demineralized dentin, and (4) decreasing the activity of endogenous hydrolytic enzymes [22].

The present study evaluated the effect of dentin pretreatment with DMSO on the microshear bond strength of a universal adhesive agent used in self and total-etch modes. According to the result, dentin pretreatment with DMSO significantly decreased the microshear bond strength of the adhesive in both self-etch and total-etch settings, and thus, the null hypothesis was rejected. In addition, the bonding technique (self-etch/ total-etch) did not significantly affect the microshear bond strength.

Several studies have evaluated DMSO potential to improve dentin-resin bonding. However, the results have been inconsistent; many studies have reported an increase in the bond strength of the adhesive to dentin [3, 8, 23–25] while few studies concluded that DMSO application had no immediate effect on the bond strength values [3, 8, 26]. It should be noted that the results of these studies might not be comparable with each other since different concentrations of DMSO and exposure times have been used in different studies. Moreover, a wide variety of adhesive systems has been employed in the previous studies which makes comparing the results even more difficult since the overall efficiency of DMSO might vary depending on the adhesive type [4]. Thus, the results of the studies which used other adhesive types than universal adhesive cannot be compared to the results of this study. We could find only two studies investigating the efficacy of DMSO application on the bond strength of a universal adhesive; Balakrishnan et al. [23] used Single Bond Universal (3M) and concluded that DMSO application increased the bond strength of the adhesive to dentin significantly. Single Bond Universal contains HEMA in contrast to G-Premio Bond used in the present study. Theoretically, monomers such as 10-MDP cover the exposed collagen fibrils in dentin in order to form a strong dentin-resin bond. However, in the presence of HEMA, HEMA molecules surround MDP molecules and reduce hydrophobic interaction between MDP molecules and collagen fibrils which in turn, decreases the bond strength of the adhesive to dentin. In the presence of DMSO, DMSO dissolves MDP and makes MDP interaction with collagen fibrils more stable [27]. This explains the increased bond strength of adhesive containing HEMA following the dentin pretreatment with DMSO. On the other hand, G-Premio Bond lacks HEMA in its composition and it might be the reason explaining why DMSO application could not increase the bond strength of G-Premio Bond as opposed to Single Bond Universal. In addition, G-Premio Bond contains acidic monomers such as 4-MET which might be negatively affected by DMSO. DMSO might interfere with the polymerization of these monomers, and subsequently, decrease the bond strength of G-Premio Bond to dentin [28]. Further studies are required to confirm this assumption. In another study which also used universal adhesive [11], DMSO application on dentin did not affect the bond strength of a universal adhesive (Scotchbond Universal) to dentin, either in self-etch or total-etch modes after 24 h and 6 months storage in water.

G-Premio Bond contains acetone as the solvent which might contribute to the decreased bond strength of this adhesive after DMSO dentin pretreatment; Szesz et al. [24] reported that the application of DMSO followed by adhesive systems containing acetone decreases the bond strength of adhesives to dentin as opposed to adhesives containing ethanol and this might contribute to the decreased bond strength of G-Premio Bondafter DMSO application.

In the present study, we used DMSO 50% (v/v) as this concentration was used in many previous studies [2, 9, 25, 29]. Salim Al-Ani et al. evaluated the effect of different concentrations of DMSO on the durability of dentin bonding and reported that after a 6-month period, the number of failures involving dentin increased in the groups in which 10% and 20% DMSO concentrations were used. They concluded that high concentrations of DMSO pretreatment might have detrimental effects on the bonding as observed in the present study. However, recently published studies have reported an increase in dentin-resin bond strength after dentin pretreatment with 50% (v/v) DMSO; Mehmood et al. [30] used 50% DMSO solution for dentin pretreatment and measured the immediate and delayed (9 months) shear bond strength of resin composite to dentin. According to their results, 50% DMSO significantly increased immediate and late shear bond strength.

Due to the dynamic nature of the oral cavity, restorations and adhesives are subjected to constant destruction by physical, mechanical, and chemical procedures. Previous studies have employed various aging procedures to simulate oral conditions including storage of samples in artificial saliva [2, 3, 25], storage of samples in distilled water [26, 31], and thermocycling procedure. This study used thermocycling for aging the samples and simulating the oral cavity environment. According to Texeira et al. [32], thermocycling decreases the bond strength of a universal adhesive to enamel and dentin significantly since thermocycling produces stress in the dentin-adhesive interface due to the different thermal expansion coefficients of adhesive and dentin. This leads to an increase in water sorption and further bond failure [33].

Regarding the etching technique, no significant difference was observed in bond strength values between self-etch and total-etch groups regardless of DMSO pretreatment. These findings are in line with the results of Yamanchi et al. [34] who reported that the bonding performances of universal adhesives were similar in self-etch and total-etch modes.

With respect to the failure mode, mixed failure was more frequent than adhesive failure in SE-C as opposed to SE-DMSO, and total etch groups in which adhesive failure was more frequent. The cohesive mode was not observed in samples. The observed mode of failure gives information on the bond quality between the adhesive and dentin. The high occurrence of adhesive and mixed failure modes is suggestive of inadequate bond in dentin-bonding interface.

Regarding the microleakage, the application of 50% DMSO and etching technique had no significant effect on the microleakage, and thus, we failed to reject the second null hypothesis. However; there was an interaction between the etching technique and DMSO application; in other words, DMSO application had different effects in total etch and self-etch modes. When DMSO was not applied on dentin, microleakage was significantly higher in samples in which adhesive was used in the self-etch mode. G-Premio bonding has a PH of 1.5. The high acidity of this adhesive produces etch patterns similar to etching with 37% phosphoric acid and produces calcium phosphate. When the adhesive is used in self-etch mode, produced calcium phosphate is not washed out by rinsing and thus, decreases the quality of the hybrid layer formed between dentin and adhesive [9]. Consequently, without the DMSO application, the level of microleakage was significantly higher compared to the total-etch mode. In contrast, when DMSO was applied to the samples, the microleakage level was not significantly different between self-etch and total-etch modes.

Regarding the effect of etching mode on the microleakage level, DMSO application had no significant effect on microleakage levels of samples in which adhesive was used in self-etch mode. These findings are in agreement with those of Stape et al. [2] who reported that application of 50% DMSO had no significant effect on microleakage levels after 24 h and 2 years. Conversely, when the adhesive was used in total-etch mode, DMSO increased microleakage. Residual solvents in the hybrid layer might result in poor quality of this layer [35]. Thus, removing excess solvents before polymerization by evaporating the adhesive solvents is recommended. The residual solvents can dilute monomers and affect the degree of conversion of the material [36]. DMSO has a low vapor pressure (about 25% of pure water in 50% concentration at 25° C) [37] and thus, cannot be evaporated completely before polymerization and dentin remains saturated by DMSO after adhesive application. The remaining DMSO can undermine the hybrid layer and affect microleakage levels. This effect was apparent in total-etch group since the samples were initially etched with 37% phosphoric acid which increased the depth of demineralization compared to self-etch samples in which phosphoric acid was not used. We speculate that due to the higher depth of demineralization in total-etch samples, remaining DMSO molecules penetrated deeper and decreased the infiltration of adhesive monomers which in turn, increased the microleakage levels. Additionally, G-premio adhesive contains acidic monomers such as MTDP, 10-MDP, and 4-MET which might have interfered with the polymerization process and decreased the degree of conversion. It should be noted that degree of conversion was not evaluated in the present study and this is one of the limitations of our work.

Previous studies have evaluated the effect of DMSO pretreatment on microleakage levels of different adhesive systems [2, 3, 8, 24, 29]. The aging procedure was performed in these studies differently. Three studies stored the samples in artificial saliva [2, 3, 8], one study stored the samples in water [24], and one study used thermocycling [29] to induce aging which is similar to the present study. In addition to the aging method, different time frames have been used in the previous studies ranging from 6 months [3] to 2 years [2]. According to the literature, 10,000 cycles of thermocycling correspond to 1 year of clinical service in the oral cavity [29]. We used 5000 cycles of thermocycling which corresponds to 6 months of function in the oral cavity. In a previous study conducted by Guo et al. [29], the effect of 50% DMSO pretreatment on the microleakage levels after 10,000 cycles of thermocycling was evaluated. According to the results, DMSO pretreatment significantly decreased the microleakage levels after thermocycling. These findings are in contrast with the findings of the present study in which DMSO pretreatment increased microleakage levels in total-etch group. Although both studies used the same concentration and application time for DMSO pretreatment, different thermocycling time and adhesive type might contribute to the contrasting results of the studies.

It should be noted that we only evaluated the microshear bond strength and microleakage levels following the application of DMSO. More studies using microindentation and Raman spectroscopy should be conducted to investigate the properties of the hybrid layer. Moreover, evaluating the effect of DMSO on the degree of polymerization of adhesives might be helpful to gain a better understanding of the DMSO mechanism of action.

The most important limitation of the present study is that although we used thermocycling to simulate the oral cavity conditions, it was impossible to thoroughly simulate the oral environment since many other factors are presented in the oral cavity that could not be simulated in the laboratory setting. In addition, we used DMSO in the ideal laboratory setting which might be slightly different from clinical settings in which saliva is present and makes tooth isolation more challenging.

Within the limitation of the present *in-vitro* study, the microshear bond strength of a universal adhesive (G-Premio Bond) to dentin decreased after dentin pretreatment with DMSO irrespective of the bonding technique (self-etch/ total-etch). Moreover, the effect of DMSO on microleakage of the dentin-adhesive interface depended on the etching technique; DMSO increased microleakage level when the adhesive was used in total-etch mode while it had no effect on the microleakage in self-etch mode. However, future studies are recommended not only to evaluate the effect of different concentrations of DMSO but also to confirm the DMSO effect on the bond strength of universal adhesives to dentin.

## Electronic supplementary material

Below is the link to the electronic supplementary material.

Supplementary Material 1

## Data Availability

All data generated or analyzed during this study are included in this published article and supplementary file 1.

## Abbreviations

DMSO: Dimethyl sulfoxide
TE: Total-etch
SE: Self-etch
C: Control
